# How Psychedelic-Assisted Treatment Works in the Bayesian Brain

**DOI:** 10.3389/fpsyt.2022.812180

**Published:** 2022-03-08

**Authors:** Daniel Villiger

**Affiliations:** ^1^Department of Psychosomatics and Psychotherapy, Psychiatric University Hospital Basel, University of Basel, Basel, Switzerland; ^2^Institute of Philosophy, University of Zurich, Zurich, Switzerland

**Keywords:** psychedelics, psychotherapy, predictive processing, common factors, REBUS hypothesis

## Abstract

Psychedelics are experiencing a renaissance in clinical research. In recent years, an increasing number of studies on psychedelic-assisted treatment have been conducted. So far, the results are promising, suggesting that this new (or rather, rediscovered) form of therapy has great potential. One particular reason for that appears to be the synergistic combination of the pharmacological and psychotherapeutic interventions in psychedelic-assisted treatment. But how exactly do these two interventions complement each other? This paper provides the first account of the interaction between pharmacological and psychological effects in psychedelic-assisted treatment. Building on the relaxed beliefs under psychedelics (REBUS) hypothesis of Carhart-Harris and Friston and the contextual model of Wampold, it argues that psychedelics amplify the common factors and thereby the remedial effects of psychotherapy. More precisely, psychedelics are assumed to attenuate the precision of high-level predictions, making them more revisable by bottom-up input. Psychotherapy constitutes an important source of such input. At best, it signalizes a safe and supportive environment (cf. setting) and induces remedial expectations (cf. set). During treatment, these signals should become incorporated when high-level predictions are revised: a process that is hypothesized to occur as a matter of course in psychotherapy but to get reinforced and accelerated under psychedelics. Ultimately, these revisions should lead to a relief of symptoms.

## Introduction

Psychedelics are on the rise again. Each of the last 5 years has included more clinical trials with psychedelics than the previous year, reaching a total of 17 trials in 2020 (1). Michael Pollan's 2018 book *How to Change Your Mind*, which covers this new science of psychedelics, became a No. 1 *New York Times* bestseller and is due to be adapted into a four-part documentary on Netflix. Thus, after several fallow decades, clinical research on and public interest in psychedelics is now flourishing.

The 1950's and 1960's constitute the first flowering of psychedelic research, with more than 1,000 papers written on the topic and with around 40,000 patients included (2). Simultaneously, and to the discontent of many researchers, psychedelics also spread beyond clinical studies and became a defining feature of that period's countercultural movement. Authorities and the public observed these developments with concern and sought to halt them. Accordingly, in 1967, the United Nations declared psychedelics a potential health and security risk and demanded strict regulation (3). The ultimate prohibition occurred 3 years later, when the United States Drug Enforcement Agency classified psychedelics as Schedule I substances, indicating that they have a high potential for dependence and no accepted medical use. This restriction was enacted despite previous research demonstrating that psychedelics are a promising therapeutic intervention and are mostly non-addictive (4). As a result of the new classification of psychedelics, more and more researchers and clinicians were banned from prescribing them, which eventually drained the entire research area (5).

Not until the end of the 20th century did psychedelics once again catch the interest of academia (6–8). While these first studies had been conducted with healthy participants, psychedelics were also examined in clinical contexts shortly thereafter [e.g., (9)]. At present, there are more than a dozen studies that investigate the therapeutic effects of psychedelics among various mental disorders such as depression, obsessive compulsive disorder (OCD), anxiety, or alcohol abuse [for a recent meta-analysis, see (10)]. And although this second flowering of psychedelic research is still in its early stages, it appears to have the potential to influence psychology and psychiatry in a major way in the coming decades.

These promising findings regarding the clinical use of psychedelics trigger the question of how they lead to such remedial effects – a question complicated by the fact that the prescription of psychedelics is normally embedded in a larger treatment, such as some form of psychotherapy. Therefore, the borders between pharmacological intervention and psychological intervention get blurred. Nevertheless, in the literature, we find explanations for the effects of psychedelics that tend to focus either on their pharmacological aspects or on their psychological aspects. The relaxed beliefs under psychedelics (REBUS) hypothesis (11) constitutes an example of the former case. It builds on the free-energy principle (12), which is closely related to hierarchical predictive processing: a theory of brain organization and functioning (13). In contrast, the common factors theory of psychotherapy (14, 15) has been used to explain the psychological aspects of psychedelic-assisted treatment (16, 17). The present paper connects these two explanatory threads. In so doing, it provides the first comprehensive account of psychedelic-assisted treatment, considering both its pharmacological and its psychological effects as well as their interaction.

The paper is structured as follows: First, a pharmacological view on psychedelic-assisted treatment introduces the REBUS hypothesis. Second, a psychological view on psychedelic-assisted treatment analyses the role of common factors in psychotherapy and describes how psychedelics fit into these common factors. Third, an integrative view on psychedelic-assisted treatment then links the previous two sections and examines how the mechanisms described in the REBUS hypothesis interact with common factors.

## A Pharmacological View on Psychedelic-Assisted Treatment

There are three drugs that are normally clustered as psychedelics: d-lysergic acid diethylamide (LSD), psilocybin, and N,N-dimethyltryptamine (DMT) (18). From a phenomenological perspective, all three lead to profound changes in perception and mood, including ego dissolution (19), near-death-like experiences (20), paranoid and delusional thinking (21), vivid autobiographical recollection (22), altered time perception (23) and more. In hindsight, such psychedelic experiences are often described as highly meaningful (24). From a pharmacological perspective, LSD, psilocybin and DMT have in common that they exert their effects mainly by serotonin 2A receptor (5-HT2AR) agonism. Accordingly, taking a 5-HT2AR antagonist before taking a psychedelic substantially attenuates its typical phenomenological effects (25).

In order to understand the neurological role of 5-HT2ARs, we have to look at how the brain is organized and thereby discuss the first theory on which the REBUS hypothesis is built: the free-energy principle (12, 26). The free-energy principle is derived from the second law of thermodynamics and provides a mathematical answer for organisms' inherent drive toward self-organization. Its basic idea involves that in order to survive, all living organisms must resist entropy, meaning self-dissolvement. This resistance is achieved by minimizing so-called free-energy. Free-energy is an information-theoretic quantity and can be thought of as the difference between the states an organism “believes” to be necessary for its adaptation, survival and reproductive success and the actual states of the organism (27). Therefore, an organism that successfully minimizes free-energy resists entropy by avoiding surprising or uncertain states, which in turn enables it to maintain homeostasis. In a nutshell, “[t]he free-energy principle furnishes a unified description of the behavior of autopoietic or living (i.e., self-producing and maintaining) systems—that explains their development, processing, and behavior based on their inherent tendency to resist disorder and minimize uncertainty” (11).

But how, exactly, is uncertainty minimized? Organisms form predictions about their environment (including themselves) and match these predictions with the incoming sensory information. This process occurs in a (approximately) Bayesian manner and results in optimizing both internal probabilistic representations of an organism's environments and how these environments are sampled (12, 28). Applied to the brain [for an application on plants, see (29)], the free-energy principle has been used to explain a variety of experimental findings including synaptic plasticity rules (30), neural prediction error signals (31), visual exploration (32, 33), neural effects of biased competition and attention (34, 35) and more [see Table 1 in (36)]. At this, it has to be mentioned that the empirical testability of the free-energy principle is still debated (37–39). Nonetheless, the Bayesian brain hypothesis has gained much traction in cognitive neuroscience in recent years (40–42), spreading the idea that the brain is not a passive stimulus-driven organ but an active probabilistic prediction machine (13, 43).

Predictive processing (also called predictive coding), which is closely related to the free-energy principle, is the most influential and best researched Bayesian approach to the brain [for a comprehensive text, see (13)]. It has received support from a vast range of theoretical and experimental studies, both with regard to primary sensory processes (44–46) and higher level cognitive processes (47, 48), such as naturalistic speech comprehension and decision making (49, 50). Furthermore, evidence has been gathered using various methods, typically with functional magnetic resonance imaging (fMRI), but also transcranial magnetic stimulation (51), electroencephalography (52–54), computational simulations (55), and physiological recordings of single neurons [cf. (56)].

Predictive processing supposes that the brain is hierarchically organized: at lower levels, predictions are highly spatially and temporally precise, whereas at higher levels, they become increasingly abstract. Across all these levels, top-down predictions should accommodate the bottom-up sensory input as efficiently as possible and therefore minimize prediction error (unaccommodated sensory input). Given that sensory input does not match predictions at some level in the hierarchy, only prediction errors (and not the whole sensory input) are sent forward to the next higher level. Generally, there are two ways in which such mismatches can then be handled. On the one hand, predictions can be adjusted to the sensory input, leading to an updating of the brain's generative model. This is called perceptual inference. On the other hand, the body can act upon its environment or itself in such a way that the consequent sensory input now matches prior predictions. This is called active inference (57). Whether there is perceptual or active inference depends on the precision (i.e., reliability) assigned to top-down predictions and the bottom-up sensory input, respectively. Here, relative and not absolute precision is decisive: the more precise the sensory input is estimated to be, relative to current predictions, the more likely perceptual inference becomes (and vice versa). From this it follows that next to accommodating sensory input, there is “a constant kind of second-order assessment (known as “precision estimation”) that determines the weighting assigned to specific predictions at all levels of processing and to different aspects of the incoming sensory signal” (58).

Physiologically, it is hypothesized that pyramidal cells play a key role with respect to precision-weighting (59). More precisely, superficial pyramidal cells are thought to pass prediction errors forward, whereas deep pyramidal cells are assumed to pass predictions downward. By increasing the postsynaptic gain or sensitivity of superficial pyramidal cells, the precision of the prediction error rises (13, 60, 61). Consequently, the precision of predictions automatically declines from a relative point of view. But is there also a way to directly modify the precision of predictions? This question brings us back to psychedelics. According to the REBUS hypothesis, psychedelics act preferentially through stimulating 5-HT2ARs on deep pyramidal cells (11). In so doing, they disinhibit or sensitize these cells, which lightens the precision of predictions. In turn, the relative precision of ascending prediction errors increases, resulting in a greater influence of bottom-up sensory input.

The densest expression of 5-HT2ARs can be found in the cortex and, here especially, in the visual cortex and high-level association regions, such as those that are part of the so-called default-mode network (DMN) (11, 62). As a result, these are the areas where psychedelics should most strongly affect the precision-weighting of top-down predictions. Here, high-level association regions and, in this way, high-level predictions seem to be of particular importance in order to understand the effects of psychedelics. There are two reasons why this is the case: First, these high-level predictions comprise our most fundamental assumptions (13). For example, the aforementioned DMN is associated with self-consciousness (63) and has been identified to be potentially the main biological substrate of the Freudian ego (64). Accordingly, if the DMN's high-level predictions (such as we are a consistent, independent entity) lose their precision, our ego and, with it, one of our most basic premises dissolve. This provides an explanation why psychedelics can lead to such surreal and ineffable experiences: by relaxing the precision of very high-level predictions, psychedelics undermine the basis of our generative model. Second, even though psychedelics also affect precision-weightings of lower- and intermediate-level predictions (e.g., coming from the primary visual cortex), higher-level predictions could still smooth out their effects. Carhart-Harris and Friston (11) exemplify this point by means of the assumption “walls don't breathe.” Even if, on an intermediate level, the comparison of sensory input and predictions suggests that a wall is breathing, there will most likely be a correction on a higher-level. This is because, on a higher level, the prediction of breathing walls has little precision compared to that of non-breathing walls. So, if there is a prediction error, it is likely to be dissolved by rejecting the prediction that the wall is breathing and by characterizing the sensory input of an apparently breathing wall as imprecise. But, of course, if the precision of higher-level predictions is attenuated as well, there might be no top-down correction of the prediction of a breathing wall. As a consequence, we actually see the wall breathing.

At this point, let's draw an interim conclusion. The REBUS hypothesis presumes that the brain is a hierarchically organized prediction machine that tries to accommodate the bottom-up sensory input and in so doing minimizes prediction error. While at lower levels in the hierarchy, predictions are spatially and temporally precise, higher-up predictions become increasingly abstract. At the highest levels, there are predictions that constitute the basis of our generative model; for example, that we have an ego (such highly abstract predictions are sometimes called *hyperpriors*). Psychedelics are hypothesized to weaken the precision of these highly abstract predictions, resulting in a greater influence of bottom-up sensory input. Ultimately, this should lead to well-known psychedelic experiences such as optical hallucinations or ego dissolution.

So far, so good. But as mentioned in the introduction, the REBUS hypothesis is grounded in two theories. The second one is the so-called entropic brain hypothesis (65, 66). It argues that “within upper and lower limits, after which consciousness may be lost, the entropy of spontaneous brain activity indexes the informational richness of conscious states” (66). Within this entropic range, there is a point of criticality which marks the transition from order to disorder. In the normal waking consciousness of healthy adult humans, the entropic state of the brain is just a bit below criticality, meaning that cognition is ordered but still somewhat flexible (67, 68). On the one hand, if entropy further decreases, we reach a state of even higher order but also higher rigidity. The entropic brain hypothesis links several mental disorders to such states: for instance, depression, OCD and addiction. Here, the brain is trapped in a dysfunctional state and cannot switch to a more functional one. On the other hand, above criticality, cognition is highly flexible but also disordered. Early psychosis is thought to involve such highly entropic states (65, 66).

What role do psychedelics play in the entropic brain hypothesis? Carhart-Harris argues that psychedelics increase the entropy of spontaneous brain activity (69–72), making the brain more flexible and interconnected and putting it closer to criticality (73–75). For example, global integration of brain regions substantially increases under psilocybin, with greater communication between regions' different communities (76). Moreover, psychedelics appear to significantly decrease prominent low-frequency rhythms such as α and β (77). Among others, these rhythms are associated with top-down functions, including inhibition and conferring top-down predictions about bottom-up sensory input (78, 79). Thus, a decrease of α and β rhythms should lead to less top-down constrained brain activity and therefore to an increase in entropy, paving the way for psychedelic effects. In line with that, the subjective intensity of psychedelic effects correlates with the decrease of α and β rhythms (77, 80).

As might have become obvious, the entropic brain hypothesis can be naturally integrated into the predictive processing account of psychedelics: a brain that is (highly) entropic is a brain where precision-weightings of top-down predictions are weakened (or generally weak), resulting in less top-down constraint and therefore greater bottom-up influence. Via stimulating 5-HT2ARs on deep pyramidal cells and thereby affecting precision-weightings of top-down predictions, psychedelics are hypothesized to produce such entropic effects on spontaneous brain activity.

From here, it is not far to the answer to the question of why psychedelics can have remedial effects on mental disorders. As mentioned above, the entropic brain hypothesis connects depression, addiction and OCD with low entropic brain states comprising high order but also high rigidity (65, 66). Accordingly, these states can hardly be overcome by making new experiences – brain activity is trapped. In predictive processing terms, the states involve overly precise high-level predictions, causing an inhibition of and insensitivity to bottom-up sensory input [for predictive processing accounts on depression, OCD and addiction, see (81–85)]. As a consequence, even in the presence of disconfirming sensory input, predictions do not get updated (11, 86). Psychedelics are believed to mitigate this rigidity by decreasing the influence of high-level predictions. In so doing, they increase the entropy of spontaneous brain activity and open the door for brain states that were previously blocked by top-down override. To summarize, the REBUS hypothesis assumes that psychedelics can vault us to totally different brain states since overly precise, dysfunctional high-level predictions are attenuated. Given the brain does not return to its rigid and dysfunctional prior states after the psychedelic experience but remains more flexible (yet, also not too flexible) due to revised high-level predictions, there should be long-term remedial effects.

To conclude the first section, let us examine the brain's state under psychedelics in more detail. Carhart-Harris and Friston (11) use the expression of the “anarchic brain” to describe it. As previously mentioned, an attenuation of high-level predictions' precision-weightings automatically leads to a greater influence of bottom-up sensory input. So, the term *anarchic* implies that bottom-up signaling is less controlled by top-down predictions and is liberated to flow upwards, impacting our perception, cognition and action more strongly. Here, normally suppressed bottom-up signaling from lower-level intrinsic systems, such as the limbic system, appears to be particularly implicated in the action of psychedelics (65, 69, 87). In turn, the disinhibition of the limbic system can explain the intense and uncontrollable emotional experiences that usually come along with taking psychedelics [cf. (88)]. Overall, it was found that the anarchic brain is more suggestible (89), more sensitive to context (90) and has elevated synaptic plasticity and efficacy (91). Therefore, the anarchic brain seems to provide perfect conditions for revisions of high-level predictions.

## A Pychological View on Psychedelic-Assisted Treatment

As the name *psychedelic-assisted treatment* suggests, the prescription of psychedelics is not an isolated process but embedded in a larger treatment, which typically is some form of psychotherapy. Therefore, when asking how psychedelics lead to remedial effects, we also have to take their psychotherapeutic surrounding into consideration. In fact, we might even go so far as to say that psychedelics themselves are a psychotherapeutic and not a psychiatric intervention. Accordingly, it is insufficient to consider only their pharmacological mechanisms of action. We also have to consider how they work from a psychotherapeutic and thus a psychological perspective.

Currently, there are more than a hundred psychotherapeutic interventions. So, it might seem imprecise to talk about psychotherapy in general. However, psychotherapeutic research has repeatedly shown that, with few exceptions (92, 93), no bona fide psychotherapy consistently outperforms another (15). It has therefore been suggested that although psychotherapeutic interventions differ in their explanatory models, they ultimately are grounded in the same common factors [for a critical assessment, see (94)]. Regarding these common factors, we mainly find three theories: the contextual model of Wampold (95), the generic model of Orlinsky (96) and the model of Frank (97) (which has no specific name).

The present paper will focus on Wampold's contextual model, as it is the most recent of the three theories and has markedly revived the debate on common factors in the last decade [for a discussion of Frank's model in the context of psychedelics, see (16)]. The contextual model consists of one prerequisite and three pathways. Having an initial therapeutic bond constitutes the prerequisite in order that psychotherapeutic work can be effective, where the key element of such a bond is trust. To be clear, trust is of course essential in all medical contexts; still, it is of particular relevance in psychotherapy. As Bordin (98) writes: “Some basic level of trust surely marks all varieties of therapeutic relationships, but when attention is directed toward the more protected recesses of inner experience, deeper bonds of trust and attachment are required and developed” (p. 254). In the most basic terms, there are two components that affect trust [cf. (15)]. On the one hand, the first encounter with the therapist is decisive. Here, the patient immediately assesses whether the therapist is trustworthy – a process that typically takes less than a second (99). Importantly, the assessment is not only based on the therapist's appearance but also on the environment (e.g., how the room is arranged and decorated). On the other hand, the patient's prior attitude toward psychotherapy is decisive too. For example, trust in psychotherapists is generally elevated if psychotherapy is a culturally accepted treatment approach (100). Moreover, patients who voluntarily and affirmatively seek therapy tend to be more trusting than patients who are socially or legally forced to undergo therapy (101).

We can assume that the prerequisite of an initial therapeutic bond is fulfilled when psychedelics are prescribed in a respective treatment since, prior to being prescribed psychedelics, the patient has already participated in multiple therapy sessions. It is well-known that the highest rate of dropout occurs immediately after the first session and then declines with each additional session (102, 103). Therefore, patients who keep up therapy until psychedelics become an option seem to perceive some value in doing so, indicating that the initial therapeutic bond has not failed (at least not completely). It might be objected that suggesting psychedelic treatment subverts the initial therapeutic bond. Indeed, even though it appears that psychedelics are slowly becoming more accepted again, there are still public reservations about their use. Many people associate psychedelics with the '68 generation, flower power and tripping hippies rather than with the scientific treatment of mental disorders. Moreover, psychedelic-friendly sectarian communities, such as the *Kirschblütengemeinschaft* founded by the Swiss psychiatrist Samuel Widmer, undermine trust in psychedelic-assisted treatments (104, 105). Nevertheless, there are good reasons why the proposal of psychedelic sessions by the therapist does not jeopardize the initial therapeutic bond. First, currently, (almost) all psychedelic-assisted treatments are part of scientific trials and are executed within university psychiatric departments, which underpins their trustworthiness. Second, the psychedelic session is usually accompanied by a male and a female clinician, which increases the level of safety [cf. (106)]. Third, the patient is free to choose whether they want to take part in one or several psychedelic sessions, with no negative consequences for their standard therapy regimen if they do not want to.

Given an initial therapeutic bond is established, three pathways for therapeutic effects open up: the real relationship, expectations and specific ingredients. The real relationship between the patient and the therapist can be seen as a continuation of the initial therapeutic bond. The idea of the real relationship has psychodynamic theoretical roots (107). It is defined by genuineness (i.e., the ability and willingness to be open, authentic and honest) and realistic perceptions (i.e., perceptions that are not distorted by transferences and other defenses). Therefore, psychotherapy should encourage and empower the patient to drop their defense mechanisms and be what they (believe that they) truly are. In order to achieve this outcome, the therapist-patient relationship has to radiate confidence, safety, support and positivity. Three key characteristics of the therapeutic setting contribute to this (95). First, with the exception of some statutory limits, interactions between patient and therapist are confidential. Second, the relationship is not disrupted by disclosure of difficult material. Third, the way the therapist interacts with the patient is empathic, appreciative and caring. Particularly, this last point seems to be of importance as “[r]atings of therapist empathy are among the most consistent predictors of psychotherapy outcome available” (15): in a recent meta-analysis, empathy ratings were substantially correlated with psychotherapy outcomes (*d* = 0.58) (108). Additionally, constructs related to empathy such as congruence (*d* = 0.46) and positive regard (*g* = 0.28) are also positively associated with psychotherapy outcomes (109, 110).

Psychedelics seem to make use of the pathway of the real relationship in a twofold way. On the one hand, there is evidence showing that psychedelics can elevate the patient's feelings of connectedness to the therapist(s). A qualitative study including patients diagnosed with depression who underwent a psilocybin session found that after the dose, many stated to feel “bonded to their guides, saying they had been through something substantial together. Rapport helped build trust that they were in safe hands and gave them a sense of an equal relationship rather than a traditional “doctor vs. patient” dyad” (111). Consequently, the real relationship appears to be strengthened by a psychedelic session. Besides, as we know from the previous section, psychedelics not only increase connectedness to the therapist but to the world in general, up to the point where the ego completely dissolves (19). On the other hand, psychedelics lead to uncontrollable experiences, which is why a common mantra when taking them is to “trust, let go, be open” (88). As rigid top-down constraints (including defense mechanisms) dismantle, new insights can be gained (11). These insights are often of high personal relevance and meaning (88, 111). Therefore, a psychedelic session might provide patients a shortcut for what psychotherapy tries to achieve via the pathway of the real relationship, namely, to enable them to be what they (believe that they) truly are.

Expectations constitute the second pathway for therapeutic effects. Here, we can differentiate two kinds of expectation: already existing expectations and expectations created by the therapy. The former include expectations such as “doing psychotherapy will have remedial effects” or “now that I have finally consulted a therapist, things will take a turn for the better” (95). Additionally, subliminal cues from the therapeutic setting can also elicit unconscious remedial expectations (15). For example, the mere fact that you are in a therapy room, doing what people do when they have mental problems, interacting with a therapist, or lying on a couch and talking about your problems might activate unconscious remedial expectations.

In addition to these existing expectations, psychotherapy also creates new expectations: for instance, by means of psychoeducation. Usually, patients come to therapy with a non-adaptive explanation for their mental problems. Here, *non-adaptive* means that the explanation does not involve a solution. Through psychoeducation, the therapist provides an alternative explanation that comprises a means to surmount, or at least to cope with, the patient's difficulties. Accordingly, the patient builds up the expectation that taking part in and “successfully completing the tasks of therapy, whatever they may be, will be helpful in coping with their problems, which then furthers for the patient the expectation that he or she has [the] ability to enact what is needed” (95). In other words, the adaptive explanation provided by the therapist is assumed to reinforce the patient's feelings of self-efficacy and mastery, which in turn should facilitate the initiation of change (112, 113).

Supporting the pathway of expectations, a recent meta-analysis of patients' pre- or early (at the end of the first session) treatment outcome expectations and their posttreatment outcomes found a positive relationship (*d* = 0.36) (114). Moreover, there is indirect evidence from research on the alliance suggesting that expectations influence treatment outcomes. The construct of the (working) alliance serves as an indicator for how much the patient embraces the therapist's adaptive explanation and the concomitant treatment (115). It involves the agreement of patient and therapist on the tasks and goals of the therapy and “the degree to which the therapy dyad is engaged in collaborative, purposive work” (116). Together with the therapist's empathy, the alliance is one of the best predictors for therapeutic success [see (95)]: a recent meta-analysis has shown that the alliance was substantially correlated with psychotherapy outcomes (*d* = 0.57) (117).

The way in which expectations (both conscious and unconscious) influence remedial effects has been extensively discussed in another research area: placebo studies (118–122). Here, placebo effects could also be found in open-label studies where participants knew that they were getting a placebo (123–125). So, deception appears not to be a requirement for eliciting placebo effects. However, if placebos are administered in an open-label setting, giving participants a rationale for why placebos work appears to be crucial (121). As both placebo and psychotherapy build on non-pharmacological and therefore psychological mechanisms, some authors have already established a connection between the two (126–128). In fact, psychotherapy itself might be a sort of (open-label) placebo, where psychoeducation delivers (part of) the rationale.

Psychedelic-assisted treatment provides a powerful basis to build up expectations. First, therapists will tell patients that previous studies have shown that it leads to promising outcomes and has great potential. Moreover, therapists prescribing psychedelics are most likely enthusiastic about this new (or, rather, rediscovered) form of treatment, as they dedicate their research and practice to it. Second, psychedelic-assisted treatment provides both a convincing pathological rationale (i.e., aberrations in the normal mechanics of hierarchical predictive processing) and treatment rationale (i.e., relaxing the precision-weighting of high-level predictions) (11). The two rationales together suggest that by undergoing a psychedelic session, patients can reduce or even overcome their mental problems (at least temporarily). Third, the psychedelic experience is embedded in preparation and integration sessions, indicating its therapeutic significance.

It has been argued that treatment effect sizes in psychedelic randomized control trials are likely over-estimated because of high levels of response expectancy (and deblinding of patients) (129). In other words, the found effects of psychedelics are hypothesized to have partly stemmed from the patients' remedial expectations about their psychedelic-assisted treatment. Additionally, a placebo study has demonstrated that a psychedelic session can induce strong expectations that are able to affect experience (130). Researchers told participants that they would receive iprocin – a homolog to psilocybin – and listed the same common effects we find in the case of psilocybin. Participants took the supposed drug in a group setting, including confederates who subtly acted out the listed effects. Results revealed that even though the drug was inert, 61% of participants verbally reported that it had some effects. Moreover, among these participants, some showed effects with magnitudes that we typically see when administering moderate or high doses of psilocybin.

The third pathway involves specific ingredients. These are the specific interventions of the respective therapy. For example, in behavioral therapy these interventions could be exposure to feared stimuli; in cognitive therapy, scrutiny of core beliefs; and in psychodynamic therapy, disclosure of unconscious inner conflicts. As previously mentioned, these specific ingredients are crucial since, by going along with them, patients get the feeling that they are doing what is necessary to overcome their difficulties. Additionally, these therapeutic actions aim to promote new behavior, insights and perception that are favorable to health (15). Ultimately, patients adopt and integrate the new behavior, insights and perception in everyday life, reinforcing recovery.

Obviously, in psychedelic-assisted treatment, the psychedelic session with its preparation and debriefing is (one of) the specific ingredient(s). In this respect, it is a very well-defined specific ingredient if we compare it to typical talk therapy: there is a clear date when psychedelics are administered (unless in blind trials with a placebo); patients take the psychedelic pill themselves, so it is a conscious act; the effects of the psychedelic pill are unmissable and non-ignorable, at least in cases of high dosage; and after some time, the psychedelic effects will have unambiguously vanished. Therefore, a psychedelic session constitutes clear therapeutic action that is well-attuned to the tasks and goals of the overall treatment: to change aberrant high-level predictions by relaxing their precision.

To summarize, the therapeutic use of psychedelics fits well into Wampold's contextual model of how psychotherapy works: they appear to strengthen the real relationship, increase remedial expectations and are a very well-defined therapeutic intervention. From this we can infer that the remedial effects of psychedelics in psychedelic-assisted therapy seem to be grounded in both pharmacological and psychological mechanisms. In the next section, we examine how these two mechanisms might interact.

## An Integrative View on Psychedelic-Assisted Treatment

The REBUS hypothesis tells us that psychedelics induce ideal conditions for revisions of high-level predictions by putting the brain in an anarchic state. But, of course, whether these revisions ultimately lead to more functional high-level predictions depends on the bottom-up signaling and thus the “environment” generating it. In the case of psychedelic-assisted treatments, psychedelic experiences are typically embedded in some form of psychotherapy. As a result, there is an inevitable interaction between the pharmacological and psychological effects of psychedelic-assisted treatment. In order to describe how these two effects might interact, we need to integrate them into a single underlying framework: predictive processing. So far, we have already discussed the pharmacological aspects of psychedelic-assisted treatment under the framework of predictive processing. But what about the psychological aspects of psychedelic-assisted treatment?

Let's first examine the common factor of the real relationship in terms of predictive processing. Humans are a eusocial species with a basic need for social connection (131). There is vast evidence in various research areas that healthy functioning depends on such social connection, whether it is called social support [e.g., (132)], belongingness [e.g., (133)], attachment [e.g., (134–136)] or lack of loneliness [e.g., (137, 138)]. Referring to the free-energy principle, we could say that being socially connected reduces free-energy and helps us to maintain homeostasis. This is because surprising or uncertain states become less likely. For example, together you can balance gains and losses, make use of positive interdependencies (139) and scrutinize your beliefs (140). In fact, one key element of humans' evolutionary success is precisely our ability to cooperate (141).

As mentioned in the previous section, the therapist-patient relationship is thought to unfold its remedial effects by radiating confidence, safety, support and positivity. Therefore, the real relationship can be assumed to make the patient's world a less uncertain place and, in this way, helps to better minimize prediction error. More precisely, the hypothesis is as follows: The psychotherapeutic setting provides a highly controllable and supportive environment that most likely differs from the patient's other past and present environments. As time goes by, the patient who is regularly exposed to this therapeutic environment gradually adapts to it. In so doing, they slowly update their high-level predictions that were formed in less controllable environments and which therefore indicate great uncertainty. Since the therapeutic environment strongly contradicts the rigid prediction of uncontrollability and uncertainty, the sensory prediction error becomes continuously larger. At some point, the sensory input signaling safety and support can no longer be explained away, which initiates an updating process.

Psychedelics appear to amplify the remedial effect of the real relationship. From a psychological perspective, we have already discussed that psychedelics tend to elevate feelings of connectedness to the therapist(s) (111). We can now form a hypothesis to explain why this is the case from a predictive-processing perspective. When taking psychedelics, the precision-weightings of our high-level predictions lighten and we become more sensitive to bottom-up sensory input. Accordingly, overly precise high-level predictions indicating uncertainty become revisable. Given there is a real relationship between a therapist and a patient, the sensory input stemming from this relationship ascends to the top of the hierarchy, accompanied by perceptual inference. Here, we should think not only of the social interactions that produce sensory input – such interactions are relatively rare in psychedelic sessions – but of the therapeutic setting more generally that does so: the patient is in a safe environment (i.e., the psychiatric clinic or therapy room), with a person they know and trust (i.e., the therapist), doing something that is well-prepared (i.e., taking psychedelics). Additionally, the softening of high-level predictions might not directly end after the psychedelic effects have worn off. For instance, Carhart-Harris and Friston (11) assume that the acquisition of new insights occurs after the acute psychedelic hot state. Therefore, the pathway of the real relationship might still be facilitated in the therapeutic debriefing of the psychedelic experience as precision-weightings of high-level predictions are still lightened. To summarize, through the psychedelic experience, the patient transfers the safety and controllability of the therapeutic environment to their generative model – a process that is hypothesized to generally occur in psychotherapy yet to get strongly accelerated under psychedelics.

In fact, the importance of the setting when taking psychedelics is an old topic in psychedelic research. Al Hubbard was one of the first who instinctively understood that the interior of sanitized hospital rooms, with their white walls and fluorescent lighting, was suboptimal for a psychedelic experience. Thus, he brought music, flowers and pictures into the treatment room to prime patients for a revelatory experience or to divert a trip when it took a bad turn (88). A few years later, Timothy Leary (142) subsumed these outward circumstances under the term *setting* and emphasized its critical relevance in shaping psychedelic experiences. Moreover, Leary paired setting with another term that is also crucial for the psychedelic experience: *the set*. The set comprises the patient's inner environments or, to put it another way, their expectations, bringing us directly to the respective common factor.

In the last section, we discussed how the pathway of expectations can have therapeutic effects and we illustrated the influence of expectations on experience by means of placebo studies. Predictive processing provides a prominent framework for how placebo effects work [e.g., (143–145)]. To put it simply, it argues that placebo effects are the product of inferential processes. From a predictive-processing perspective, having an expectation is equivalent to having a top-down prediction. So, we incorporate the bottom-up input signaling remedial effects into our generative model via perceptual inference. The more precise we assess this new prediction to be, the more likely prediction error gets minimized *via* active inference. So, a highly precise prediction of remedial effects makes us act on our outer and inner world such that the prediction becomes real (145). For example, if a person strongly predicts that some inert drug reduces their pain, an endogenous opioid release might lead to the expected effect (146). More precisely, the process of active interference minimizes the prediction error that results from the bottom-up input of taking an inert pill and the top-down prediction of taking a pharmacological substance that reduces pain: because there is no exogenous opioid release from the drug that has been taken, the brain produces an endogenous one to match the prediction [for a meta-analysis on the neural correlates of placebo analgesia, see (147)].

If we apply the predictive processing framework for placebo effects to the psychotherapeutic pathway of expectations, we attain the following hypothesized mechanism. By inducing top-down predictions of amelioration and recovery, psychotherapy initiates inferential processes. Small changes of interoceptive input are no longer interpreted as mere noise but as a sign of recovery, leading to a consequent experience of relief. So here, dysfunctional high-level predictions causing distress are superseded by induced predictions of remedy that involve a different sampling of input sources. Additionally, highly precise predictions of recovery launch a self-fulfilling prophecy and thereby act on the body and its environment in such a way that they come true. To give a simplified example: because of psychoeducation, a depressive patient starts to believe that their difficulties are surmountable. This revives or reinforces their confidence, enabling them to (partly) free themselves from their inactivity. In turn, by interacting with the world, they find that it becomes more controllable and thereby less uncertain, reducing depressive symptoms (81, 148). Therefore, active inference leading to explorative behavior ultimately fulfills the prediction that the patient's difficulties are surmountable or at least to some degree manageable.

How may psychedelics interact with the pathway of expectations? Under psychedelics, the brain becomes more suggestible (89). This is assumed to occur because high-level predictions no longer constrain the lower levels of the hierarchy significantly. Linked to this, synaptic plasticity becomes elevated, which is hypothesized to facilitate the updating processes (91). As a consequence, induced expectations that have been (partly) suppressed by contrasting higher-level predictions should now be able to flow up the hierarchy and alter the generative model. Here, it seems important to differentiate two phases: the acute psychedelic hot state and the cooling-off state. In the case of the former, the patient has little or no control over the experience, which includes intense emotions stemming from a disinhibited limbic system (11). Still, these emotions might to some degree be primed by the preceding preparatory session(s). If the patient starts the psychedelic journey with a positive basic feeling and attitude, believing that whatever will happen will be good for them, this might become the undertone of the whole experience. In this way, their positive basic feeling and attitude should be amplified under psychedelics and thereby incorporated into their generative model. After the acute psychedelic hot state, we enter a different phase. The patient is back in control and has to integrate the psychedelic experience into their life. At this juncture, the patient's expectations about the role and effects of a psychedelic session in the respective treatment are, once again, essential for how to interpret their experiences. If patients believe that the psychedelic session has remedial effects, they are more likely to spin their interpretation of the experience in that direction. This verbal interpretation (in fact, language in general), whether it is self-produced, encountered or conversationally co-constructed, is hypothesized to function as a tool for manipulating precision-weightings [cf. (13)]. Therefore, the way a patient interprets and thereby integrates their psychedelic experience is thought to lead to respective adjustments of their generative model, which might still be prone to revision. Altogether, we can conclude that psychedelics may boost the pathway of expectation for at least two reasons: (1) as they are assumed to induce a state where high-level predictions become revisable, bottom-up signals primed by remedial expectations should become more influential; and (2) they produce an unreal and ineffable experience that provides immense scope for interpretation and thus the possibility to find what we want to find.

The last paragraph suggests that psychedelics (to some degree) give us what we expect to get from them – similar to a placebo. This is not a new idea. Weil (149) described psychedelics as a kind of active placebo: while they certainly do something, most of what that is may come from the consumers themselves. Grof (22) argues that “psychedelics function more or less as non-specific catalysts and amplifiers of the psyche” (p. 11). Finally, Matthew Johnson says about psychedelic treatment: “Whatever we're delving into here, it's in the same realm as the placebo. But a placebo on rocket boosters” (88). As we know from the previous section, psychotherapy has also been described as a placebo (an open-label one). Accordingly, we may say that a psychedelic session boosts the placebogenic effects of psychotherapy, making psychedelic-assisted treatment a kind of super placebo.

Let's summarize the hypothesized interaction between psychological and pharmacological effects in psychedelic-assisted therapy. Psychotherapy (co-)defines a patient's set and setting, meaning their inner and outer environments: it induces remedial expectations and provides a safe surrounding. When the brain enters an anarchic state because of the assumed effects of psychedelics, the bottom-up signals sent from these inner and outer environments become more influential. In this way, the patient should be able to transfer the safety and controllability of the therapeutic environment to their generative model, boosting the pathway of the real relationship. Similarly, psychedelics appear to boost the pathway of expectations as well. On the one hand, the impact of remedial expectations is hypothesized to be less restrained by higher-level predictions. Any positive basic feeling and attitude that a patient may have toward undergoing a psychedelic experience tends to be amplified under psychedelics. On the other hand, when integrating the psychedelic experience into their life, the patient has sufficient room for interpretation. So, if they start a psychedelic session with remedial expectations, it is likely that they will find remedial cues when interpreting it later. Overall, we can assume that the pharmacological effects of psychedelics reinforce and accelerate the psychological effects of psychotherapy.

Three final notes: First, the attentive reader might have noticed that this section did not discuss the last of the three pathways, namely the pathway of specific ingredients. The following reason clarifies this approach: psychedelics *are* a specific ingredient of psychedelic-assisted treatment. So here, we do not *per se* have an interaction between pharmacological and psychological effects. Instead, psychedelics with their pharmacological effects are part of the treatment and its rationale and thereby become vehicles for psychological effects.

Second, it has to be emphasized that the REBUS hypothesis and the theories it builds on (i.e., the free-energy principle, predictive processing and the entropic brain hypothesis) are controversial. (1) Although Carhart-Harris and Friston (11) present various indirect evidence for the REBUS hypothesis and further indirect and more direct evidence has recently been published (150, 151) or is likely to be published soon (152), there are also observations that seem to count against the REBUS hypothesis. For instance, if psychedelics decrease prediction capacity due to weakened precision of high-level predictions, prediction errors should increase. Yet, evidence for this has been mixed (153–155). Or LSD has been shown to not decrease but increase top-down influence in some regions, namely from the parahippocampal gyrus (seen as nearly unequivocally high level) to visual cortex (seen as nearly unequivocally lower level) (156). (2) As previously mentioned, the free-energy principle might not be an empirically testable theory but rather a mathematical principle (which according to Hohwy (39) is not *per se* a problem). (3) The presence of cognitive biases [e.g., (157, 158)] seems to cast doubt on the assumption of a Bayesian brain (159) (but Tappin and Gadsby (160) provide a Bayesian defense against these doubts). (4) An extensive evaluation of neurophysiological evidence for predictive processing as a model of perception has found mixed support for predictive processing's key hypotheses (161) (but the authors note that there is much work yet to be done and it is striking that clear-cut counterevidence has yet to emerge). (5) Carhart-Harris (66) himself describes the entropic brain hypothesis as speculative and forward looking, making it vulnerable to critique [e.g., (162)] (but he also argues that the major arguments and hypotheses of the original entropic brain paper have stood up well to empirical scrutiny). So, we see that the theories on which the present paper builds are not undisputed. At this, it is important to note that the purpose of the present paper is not to defend these theories (although I find them promising and prima facie convincing). Instead, the purpose of the present paper is to explore how psychedelic-assisted treatment (and not just psychedelics) works in the Bayesian brain, assuming that the REBUS hypothesis is valid. In so doing, it assimilates the REBUS hypothesis and the contextual model into an integrative account and formulates new assumptions that are speculative but testable by future research (see next paragraph).

Third, the following empirical hypotheses can be derived from the present paper: expectations about the psychedelic session should correlate with its therapeutic success [for a study that reaches this conclusion in a non-therapeutic context with microdosing, see (163)]; psychedelic sessions that occur in a familiar therapeutic environment which patients perceive as safe and controllable are more effective; the real relationship between therapist and patient (e.g., measured via ratings of therapist empathy) is predictive for the therapeutic success of a psychedelic session; the alliance (116) is predictive for the therapeutic success of a psychedelic session; and providing patients a pathological rationale (i.e., aberrations in the normal mechanics of hierarchical predictive processing) and a treatment rationale (i.e., relaxing the precision-weighting of high-level predictions) increases the therapeutic effects of a psychedelic session.

## Conclusion

In psychedelic-assisted treatment, the pharmacological intervention and the psychotherapeutic intervention appear to work hand in hand. By stimulating 5-HT2ARs on deep pyramidal cells, psychedelics are hypothesized to lighten the precision of high-level predictions, making them more prone to revision by the upwards flowing sensory input. Therefore, rigid, dysfunctional high-level predictions that are believed to lie at the core of mental disorders become revisable. In turn, the psychotherapeutic intervention is an essential source from which the bottom-up sensory input proceeds. Basically, it seems that psychotherapy unfolds its remedial effects by establishing a real relationship, inducing remedial expectations and implementing specific ingredients. Under psychedelics and the assumed anarchic brain states they cause, these pathways should become highways. Signals of safety and controllability stemming from the real relationship, and from the therapeutic environment generally, should become more easily incorporated into the patient's generative model. Additionally, the remedial effects of respective expectations should be less blocked by contradicting high-level predictions. Besides, regarding the treatment as a whole, the psychedelic experience itself constitutes a well-defined specific ingredient that creates remedial expectations and appears to intensify the real relationship.

Sixty years ago, the influence of set and setting on psychedelic experiences had already been understood to be significant. This function of being a non-specific amplifier of what is already there seems to precisely be what makes psychedelics and psychotherapy a promising combination. In psychedelic-assisted treatment, the psychotherapeutic intervention substantially co-defines the set and setting of the pharmacological intervention. Therefore, to borrow the expression from Matthew Johnson, psychedelics appear to turn psychotherapy into an intervention on rocket boosters.

## Data Availability Statement

The original contributions presented in the study are included in the article/supplementary material, further inquiries can be directed to the corresponding author.

## Author Contributions

The author confirms being the sole contributor of this work and has approved it for publication.

## Funding

This research was funded by the Swiss National Science Foundation grant number 186151.

## Conflict of Interest

The author declares that the research was conducted in the absence of any commercial or financial relationships that could be construed as a potential conflict of interest.

## Publisher's Note

All claims expressed in this article are solely those of the authors and do not necessarily represent those of their affiliated organizations, or those of the publisher, the editors and the reviewers. Any product that may be evaluated in this article, or claim that may be made by its manufacturer, is not guaranteed or endorsed by the publisher.
